# MiR-424 and miR-27a increase TRAIL sensitivity of acute myeloid leukemia by targeting PLAG1

**DOI:** 10.18632/oncotarget.8252

**Published:** 2016-03-22

**Authors:** Yan-ping Sun, Fei Lu, Xiao-yu Han, Min Ji, Ying Zhou, A-min Zhang, Hong-chun Wang, Dao-xin Ma, Chun-yan Ji

**Affiliations:** ^1^ Department of Hematology, Qilu Hospital, Shandong University, Jinan 250012, China

**Keywords:** miR-424, miR-27a, PLAG1, TRAIL resistance, acute myeloid leukemia

## Abstract

Although microRNAs have been elaborated to participate in various physiological and pathological processes, their functions in TRAIL resistance of acute myeloid leukemia (AML) remain obscure. In this study, we detected relatively lower expression levels of miR-424&27a in TRAIL-resistant and semi-resistant AML cell lines as well as newly diagnosed patient samples. Overexpression of miR-424&27a, by targeting the 3′UTR of PLAG1, enhanced TRAIL sensitivity in AML cells. Correspondingly, knockdown of PLAG1 sensitized AML cells to TRAIL-induced apoptosis and proliferation inhibition. We further found that PLAG1 as a transcription factor could reinforce Bcl2 promoter activity, causing its upregulation at the mRNA level. Both downregulated PLAG1 and elevated expression of miR-424&27a led to Bcl2 downregulation and augmented cleavage of Caspase8, Caspase3 and PARP in the presence of TRAIL. Restoration of Bcl2 could eliminate their effects on AML TRAIL sensitization. Overall, we propose that miR-424&27a and/or PLAG1 might serve as novel therapeutic targets in AML TRAIL therapy.

## INTRODUCTION

Although advances have been achieved in stem-cell transplantation and standard chemotherapy of acute myeloid leukemia (AML) over the past few decades, chemoresistance and therapy-related mortality still play a vital role in therapeutic failure and poor outcomes [1–3]. TNF-related apoptosis-inducing ligand or Apo2L (Apo2L/TRAIL), a member of the TNF family expressed mainly by cells of the immune system, is able to trigger ‘extrinsic and intrinsic apoptosis’ that specifically kill tumor cells, rendering it an anti-tumor agent of great value [4]. However, the clinical use of TRAIL for cancer therapy is now limited since quite a few human cancer cells, such as leukemia cells, are resistant to TRAIL-induced apoptosis either primarily or aquiredly [5–7]. Many researches have addressed this issue and put forward several possible mechanisms including the non-functional binding to death decoy receptor 1 or 2 (DcR1, 2), reduced Caspase expression, dysfunctions of death receptor 4 (DR4) and death receptor 5 (DR5) or overexpression of anti-apoptotic proteins cellular FLICE inhibitory protein (cFLIP), Mcl-1, Bcl2, etc [8, 9]. Still a large portion of TRAIL resistance awaits to be unveiled.

MicroRNAs (miRNAs) are a large group of small non-coding RNAs that negatively regulate the expression of target genes by binding to the 3′ untranslated region (UTR) of their mRNAs, resulting in translation inhibition or mRNA degradation [10]. To date, tremendous studies have expounded the versatile roles that miRNAs play in proliferation [11], metastasis [12], differentiation [13], apoptosis, hematopoiesis [14], angiogenesis and carcinogenesis [15]. Meanwhile aberrant expression of miRNAs has been constantly identified in pathogenesis and chemoresistance of hematopoietic malignancies. Overexpression of miR-126 was associated with poor prognosis in a large cohort of older cytogenetically abnormal AML patients and participated in leukemia stem cells long-term maintenance and self-renewal [16]. Reduced *in vitro* leukemic colony forming ability and attenuated *in vivo* leukemia-initiating cell activity of Hox-based leukemias were caused by therapeutic inhibition of miR-21 and miR-196b [17]. Chen Y et al. reported that CXCR4 induces AML chemoresistance by downregulating let-7a [18]. Moreover, some researches lately have shown that miRNAs are also involved in TRAIL resistance of cancer cells. Compared with less invasive and/or normal lung and liver cells, miR-221&222 were overexpressed in aggressive non-small cell lung cancer and hepatocarcinoma, which facilitated their functions on inducing TRAIL resistance by targeting PTEN and TIMP3 tumor suppressors [19]. These data indicate that miRNAs exerted effects on both AML development and TRAIL resistance of tumors. Whether they influence AML TRAIL resistance remains unknown.

MiR-424 has recently been classified into a large cluster family together with miR-15/miR-16, members of which generally considered as tumor suppressors by inhibiting proliferation and promoting apoptosis [20]. Decreased expression of miR-424 was observed in the hepatocellular carcinoma tissues compared with that of the normal liver tissues and correlated with higher pathological grades and more advanced TNM stages [21, 22]. Downregulation of miR-424 in treatment-naive chronic lymphocytic leukemia (CLL) patients due to epigenetic transcriptional silencing resulted in overexpression of the oncogenic transcription factor pleomorphic adenoma gene 1 (PLAG1) [23]. Meanwhile, miR-27a was reported to act as a tumor suppressor in acute leukemia cells at least in part by promoting apoptosis via 14-3-3θ regulation [24]. Although mounting evidence indicate that miR-27a contributes to chemoresistance in tumors [25, 26], it seems to have distinctive biological behaviors in AML drug resistance. However, very limited information is available concerning miR-424 and miR-27a involvement in AML TRAIL resistance.

In this study, we demonstrated that miR-424&27a were expressed at significantly lower levels in TRAIL-resistant (K562) and semi-resistant AML cell lines (HL-60, NB4) compared with sensitive AML cells (HL-60/ADM). We then presented evidence that overexpression of miR-424&27a led to elevated TRAIL sensitivity. Afterwards we identified the common target gene PLAG1 of miR-424&27a, which was verified by luciferase assays. Knockdown of PLAG1 increased TRAIL-induced apoptosis independently with lowered Bcl2 expression and potentiated cleavage of Caspase8, Caspase3 and PARP. Notably Bcl2 was previously predicted to be PLAG1 downstream target. We further confirmed that PLAG1 expression was positively correlated with Bcl2 in AML cell lines and newly diagnosed AML patients. Moreover, ectopic PLAG1 could enhance Bcl2 promoter activity and thus upregulate its mRNA expression. Collectively, we proposed that upregulation of either miR-424 or miR-27a would improve TRAIL sensitivity of AML cells by transcriptional repression of Bcl2 through directly targeting the 3′UTR region of PLAG1.

## RESULTS

### Decreased expression of miR-424 and miR-27a in TRAIL-resistant AML cells

**Figure 1:**
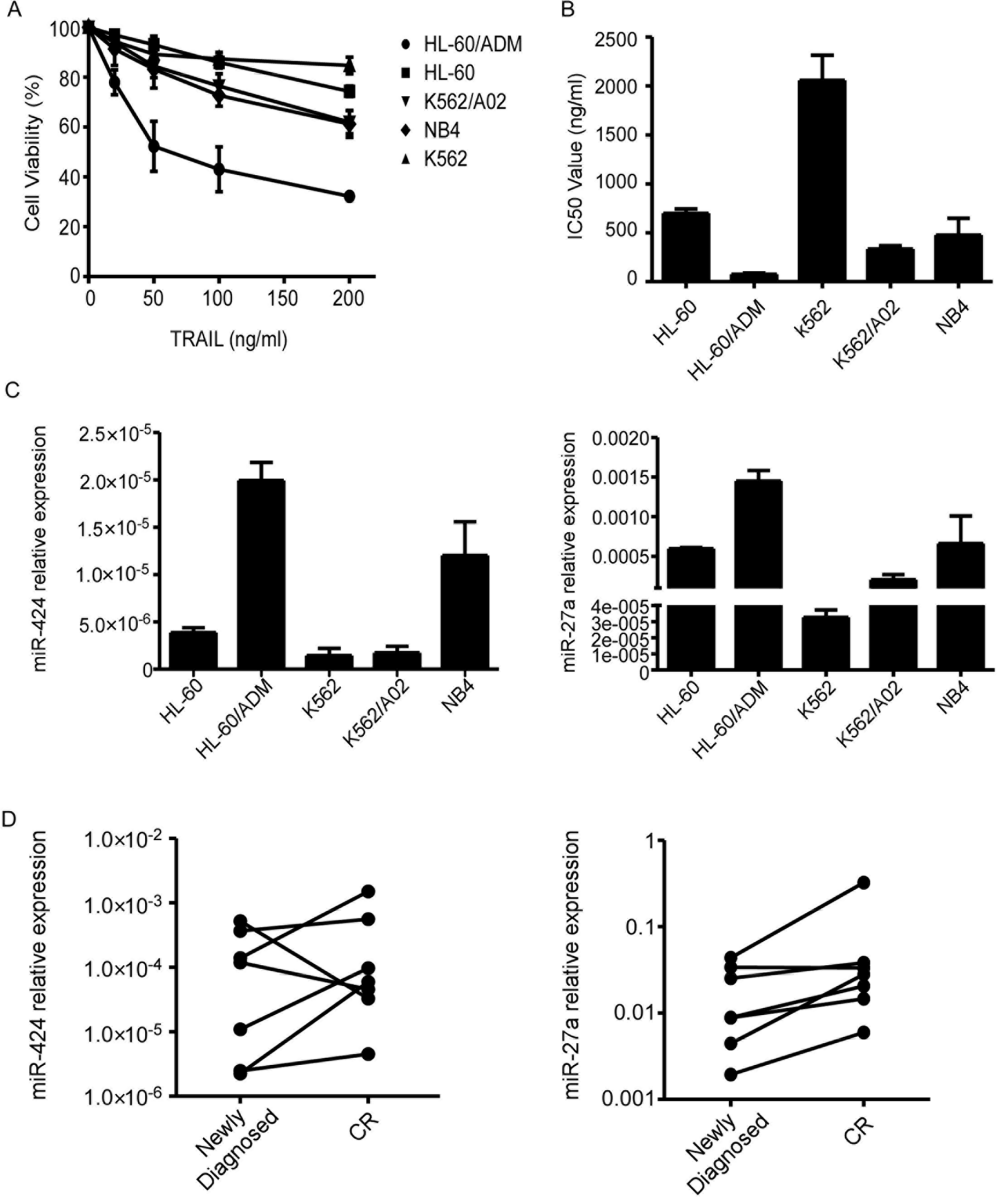
MiR-424 and miR-27a expression in AML cell lines and bone marrow blasts from AML patients (**A**) Relative survival of each AML cell line was tested with CCK8 assay after 48 h treatment with TRAIL. Points are the average of three independent experiments; error bars represent S.E. (**B**) IC50 values of AML cell lines. Columns indicate the means of triplicate results and bars represent S.E. (**C**) Real time RT-PCR of miR-424 and miR-27a expression in AML cells. The relative expression of miRNAs was normalized to U6. (**D**) Expression levels of miR-424 and miR-27a in the BM samples from most AML patients increased when they achieved complete remission compared with that of the samples from the same patients at the time of diagnosis.

To analyse TRAIL sensitivity of different human AML cell lines, we performed proliferation assays and calculated the IC_50_ values. The IC_50_ values of HL-60, HL-60/ADM, K562, K562/A02 and NB4 cells were 699.34 ng/ml, 76.71 ng/ml, 2057.05 ng/ml, 333.70 ng/ml and 476.33 ng/ml respectively (Figure 1A–1B). Based on the above results, we classified AML cell lines into TRAIL-resistant (K562), semi-resistant (HL-60, NB4, K562/A02) and TRAIL-sensitive (HL-60/ADM) groups. We chose TRAIL-resistant K562 as well as semi-resistant HL-60 and NB4 cells as the research objects to investigate the molecular mechanisms underlying AML TRAIL resistance.

To clarify the specific roles that miRNAs play in AML TRAIL resistance, we first detected miR-424 and miR-27a expression in AML cell lines. As shown in Figure 1C, miR-424 and miR-27a were markedly downregulated in TRAIL-resistant cells while upregulated in TRAIL-sensitive cells. Semi-resistant HL-60 and NB4 exhibited intermediate levels of miR-424 and miR-27a expression. Eventhough K562/A02 was semi-resistant to TRAIL, its miR-424 and miR-27a expression levels were only slightly higher than that of K562. These results suggest that decreased expression of miR-424 and miR-27a may contribute to TRAIL resistance of AML cells.

Additionally, upregulation of miR-424 and miR-27a was observed in AML patients when they attained complete remission (CR) in contrast to the that of same patients at the time of diagnosis (Figure 1D). MiR-424 and miR-27a may also serve as novel biomarkers for AML pathogenesis.

### Roles of miR-424 and miR-27a in AML TRAIL resistance

**Figure 2:**
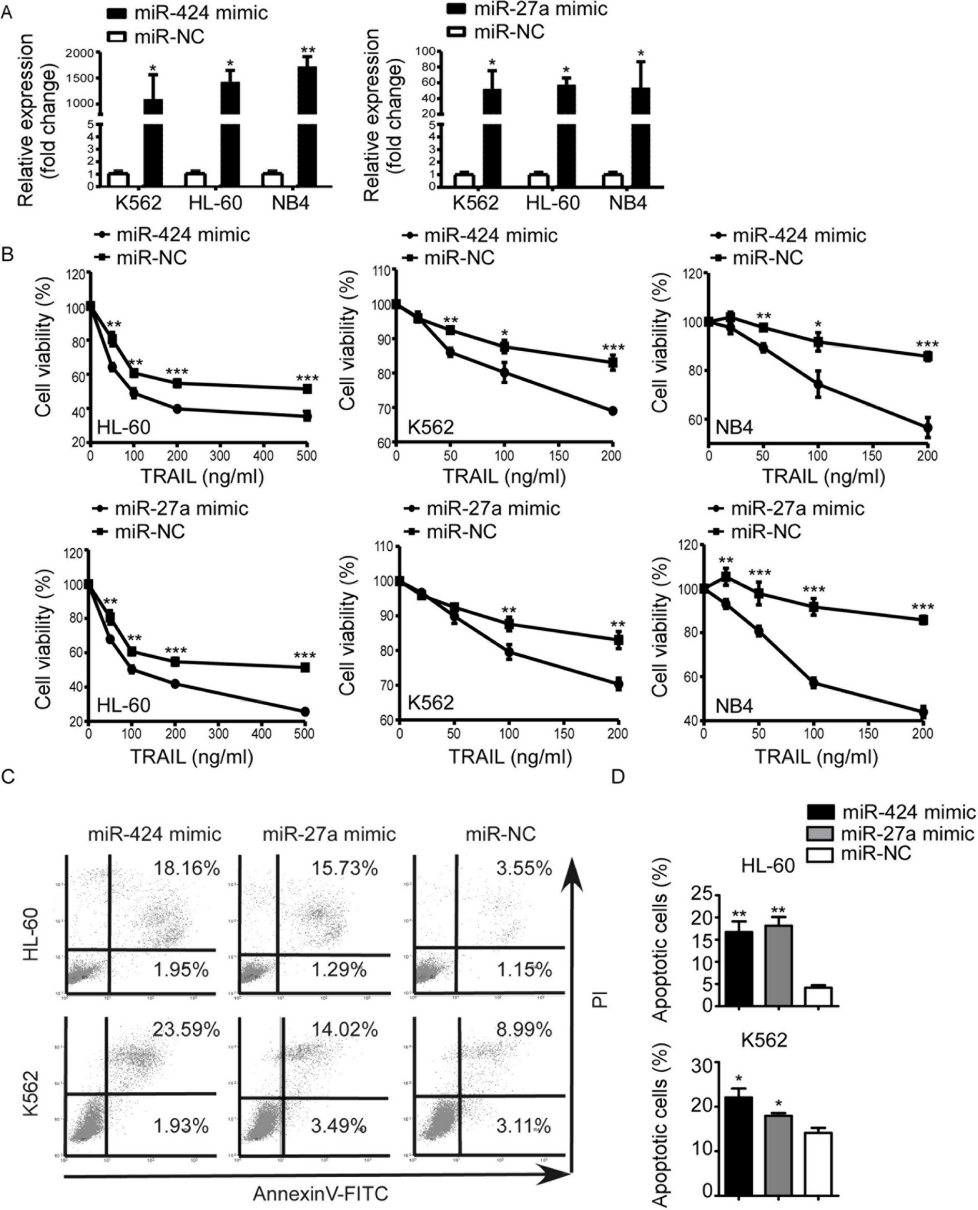
Upregulation of miR-424 and miR-27a enhanced AML sensitivity to TRAIL K562, HL-60 and NB4 cells were transfected with miR-424 mimic, miR-27a mimic or negative control (NC) for 48 h. (**A**) Expression level of miR-424 and miR-27a was measured by qRT-PCR 48 h after transfection. (**B**) Proliferation of each AML cell line after 48 h of transfection was assessed by CCK8 assay treated with different doses of TRAIL. It was plotted as a percentage of change relative to the control. *p < 0.05, **p < 0.01, ***p < 0.001. (**C**) AnnexinV/PI assays in HL-60 (above panel) and K562 (bottom panel) cells after 48 h of transfection with miR-424 mimic, miR-27a mimic or NC in the presence of TRAIL for 48 h. A representative experiment for each cell line is shown. (**D**) The proportions of apoptotic cells measured by AnnexinV/PI assays were quantified. Columns indicate means of three independent determinations, bars represent S.E. *p < 0.05, **p < 0.01.

To further demonstrate the functional association between miR-424&27a downregulation and AML TRAIL resistance, we transiently transfected NB4, HL-60, and K562 cells with either miR-424 or miR-27a mimic and scrambled control. Increased expression of miR-424 and miR-27a upon transfection were confirmed by qRT-PCR (Figure 2A). After 48 h of transfection, proliferation assays showed that overexpression of miR-424 and miR-27a both led to enhanced inhibition of proliferation in comparison with controls (Figure 2B). Apoptosis assays demonstrated that enforced miR-424 and miR-27a expression raised the proportion of apoptotic cells evidently with regard to their respective controls. These results indicated that upregulation of miR-424 and miR-27a restored TRAIL sensitivity.

### MiR-424 and miR-27a both target PLAG1 3′UTR directly

**Figure 3:**
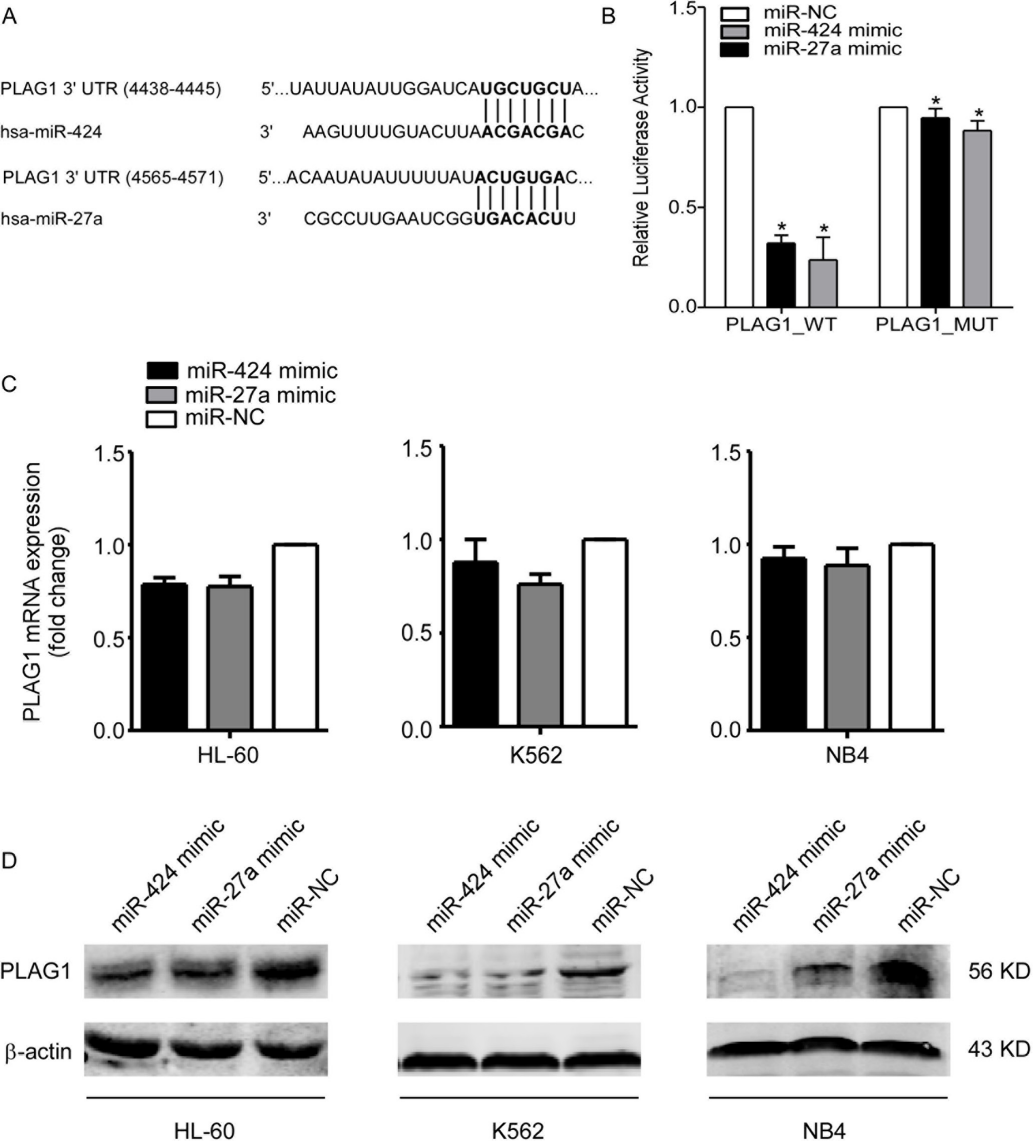
MiR-424 and miR-27a target PLAG1 3′UTR directly (**A**) TargetScan predicted combination sites of miR-424 and miR-27a respectively in the 3′UTR of PLAG1. (**B**) A dual luciferase assay conducted in 293T cells cotransfected with pPLAG1_WT, pPLAG1_424M or pPLAG1_27aM and either miR-424 mimic, miR-27a mimic or a scrambled control. Luciferase activities were calculated as a ratio of firefly to renilla luciferase activity and are expressed as means ± S.E.s of three independent experiments. *p < 0.05. (**C**) qRT-PCR of PLAG1 mRNA expression in HL-60, K562 and NB4 cells after 48 h of transfection with either miR-424 mimic, miR-27a mimic or a scrambled control. We used GAPDH as a normalization control. Columns represent means ± S.E.s from three separate experiments. (**D**) Western Blot analysis of PLAG1 protein expression in HL-60, K562 and NB4 cells after 48 h of transfection with either miR-424 mimic, miR-27a mimic or a scrambled control. The protein loading controls were performed using β-actin.

Given the fact that miR-424 and miR-27a could ameliorate TRAIL resistance of AML cells, we adopted measures to unveil the underlying molecular mechanisms of this phenomenon. First of all, we used bioinformatic methods (TargetScan, Pictar) to predict potential targets of miR-424 and miR-27a. Among the candidate targets, 3′UTR of human PLAG1 (NM_001114634) contained regions (nucleotides 5218–5225; 4565–4571) that matched the seed sequences of miR-424 and miR-27a respectively (Figure 3A). Luciferase reporter assays showed that transfection of 293T cells with miR-424 or miR-27a mimic reduced the pPLAG1_WT luciferase activity by 4.21-fold and 3.11-fold respectively. This repression could be undermined with mutations at the predicted binding sites, suggesting that miR-424 and miR-27a were able to specifically target the putative regions in the 3′UTR of PLAG1 (Figure 3B).

After being transfected with miR-424 or miR-27a mimic for 48 h, PLAG1 expression of AML cells was examined at both mRNA and protein levels. The results presented that mRNA expression was scarcely influenced (Figure 3C) whereas endogenous PLAG1 protein levels of AML cell lines had been significantly downregulated (Figure 3D). All these data suggested that PLAG1 is a direct target of both miR-424 and miR-27a.

### Downregulation of PLAG1 increased TRAIL sensitivity of AML cells

**Figure 4:**
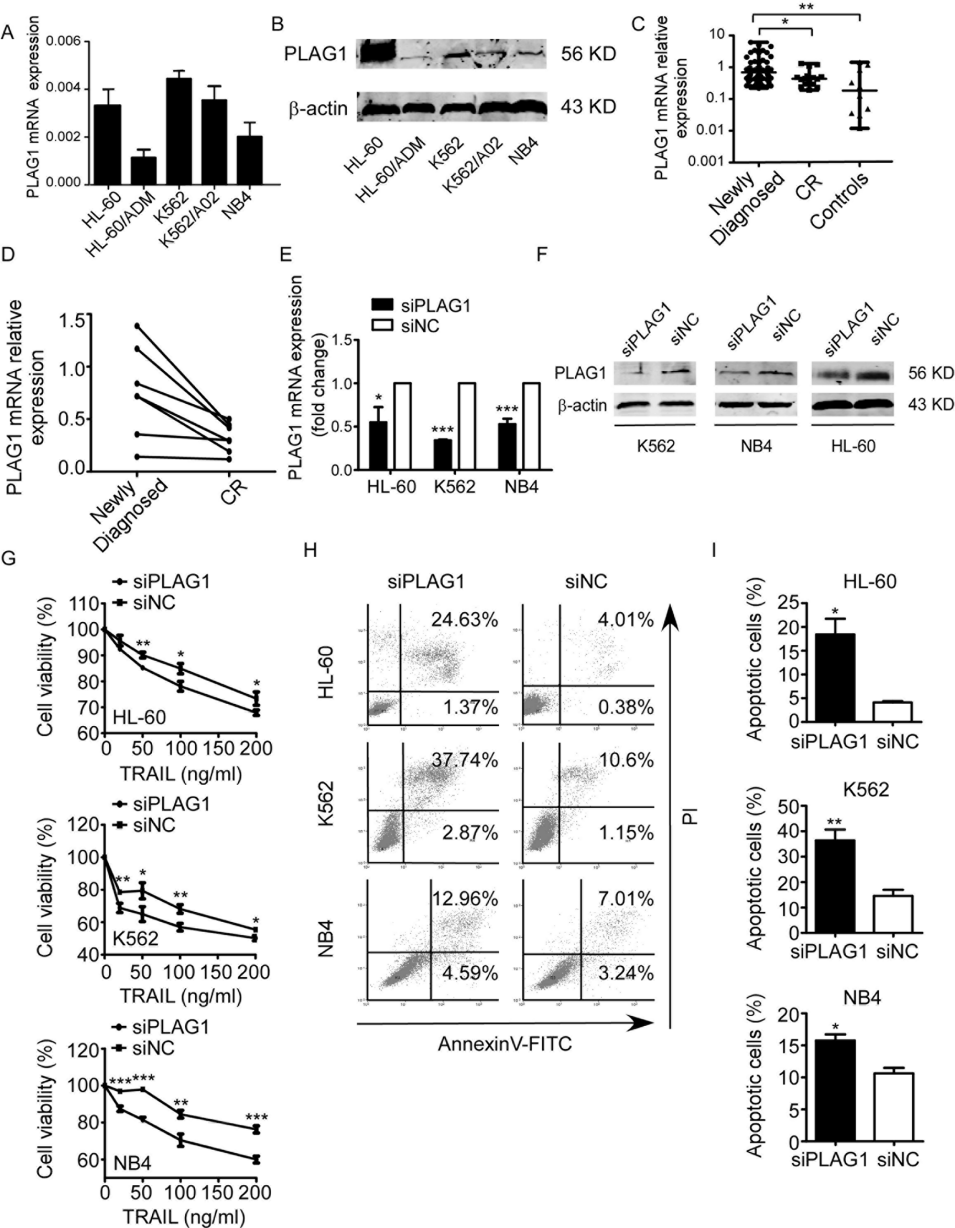
Decreased expression of PLAG1 increased TRAIL sensitivity of AML cells (**A**) qRT-PCR of PLAG1 mRNA expression in AML cell lines. (**B**) Western Blot analysis of PLAG1 protein expression in AML cell lines. (**C**) qRT-PCR detected expression of PLAG1 in 62 newly diagnosed AML patients, 16 CR patients and 10 controls. Solid points indicate individual values and horizontal lines represent medians with range. *p < 0.05, **p < 0.01. (**D**) qRT-PCR demonstrated that PLAG1 mRNA expression in the BM samples decreased when AML patients achieved complete remission compared with that of the samples obtained from the same patients at the time of diagnosis. (**E–F**) qRT-PCR and Western Blot analysis verified the downregulation of PLAG1 in AML cells after 48 h of transfection with siPLAG1.*p < 0.05, ***p < 0.001. (**G**) CCK8 assays were conducted after 48 h of transfection with siPLAG1. AML cells proliferation was inhibited by PLAG1 silencing with supplement of different concentrations of TRAIL. Points are the average of three independent experiments; error bars represent S.E.; *p < 0.05, **p <0.01, ***p < 0.001. (**H**) AnnexinV/PI assays in HL-60 (above panel), K562 (medium panel) and NB4 (bottom panel) cells after 48 h of transfection with siPLAG1 treated by 200 ng/ml TRAIL for 48 h. A representative experiment for each cell line is shown. (**I**) The proportions of apoptotic cells calculated from triplicate experiments increased after PLAG1 downregulation compared with controls. *p < 0.05, **p < 0.01.

We continued to investigate whether PLAG1 contributed to TRAIL resistance of AML cells. PLAG1 upregulation was observed in TRAIL-resistant (K562) and semi-resistant (HL-60 and NB4), versus TRAIL-sensitive cells (HL-60/ADM) (Figure 4A). Despite of the moderate sensitivity to TRAIL, K562/A02 possessed PLAG1 expression level inferior only to K562 (Figure 4A). By Western Blot analysis, we discovered that endogenous PLAG1 protein level of TRAIL-resistant and semi-resistant cells was distinguishably higher than that of the TRAIL-sensitive cells (Figure 4B). Next, we silenced PLAG1 with siRNA, which prominently reduced endogenous PLAG1 expression (Figure 4E–4F). After 48 h exposure to TRAIL, proliferation and apoptosis assays were conducted. AML cells with PLAG1 knockdown showed a much lower proliferation rate compared with the control groups (Figure 4G). AnnexinV-FITC assays on TRAIL-resistant and semi-resistant cell lines revealed an significant increase in TRAIL-induced apoptosis after PLAG1 silencing (Figure 4H–4I).

To better illustrate the specific roles that PLAG1 plays *in vivo*, we assessed its mRNA expression in AML patients as well as controls. PLAG1 was notably overexpressed in newly diagnosed patients with respect to either CR patients or controls (Figure 4C). In addition, BM samples obtained from the same patients at both the newly diagnosed and the CR state were analyzed for their PLAG1 expression. Most patients exhibited an sharp decrease in PLAG1 mRNA level once they achieved the CR state (Figure 4D).

### MiR-424 and miR-27a downregulated Bcl2 and potentiated cleavage of Caspase8, Caspase3 and PARP through modulating PLAG1

**Figure 5:**
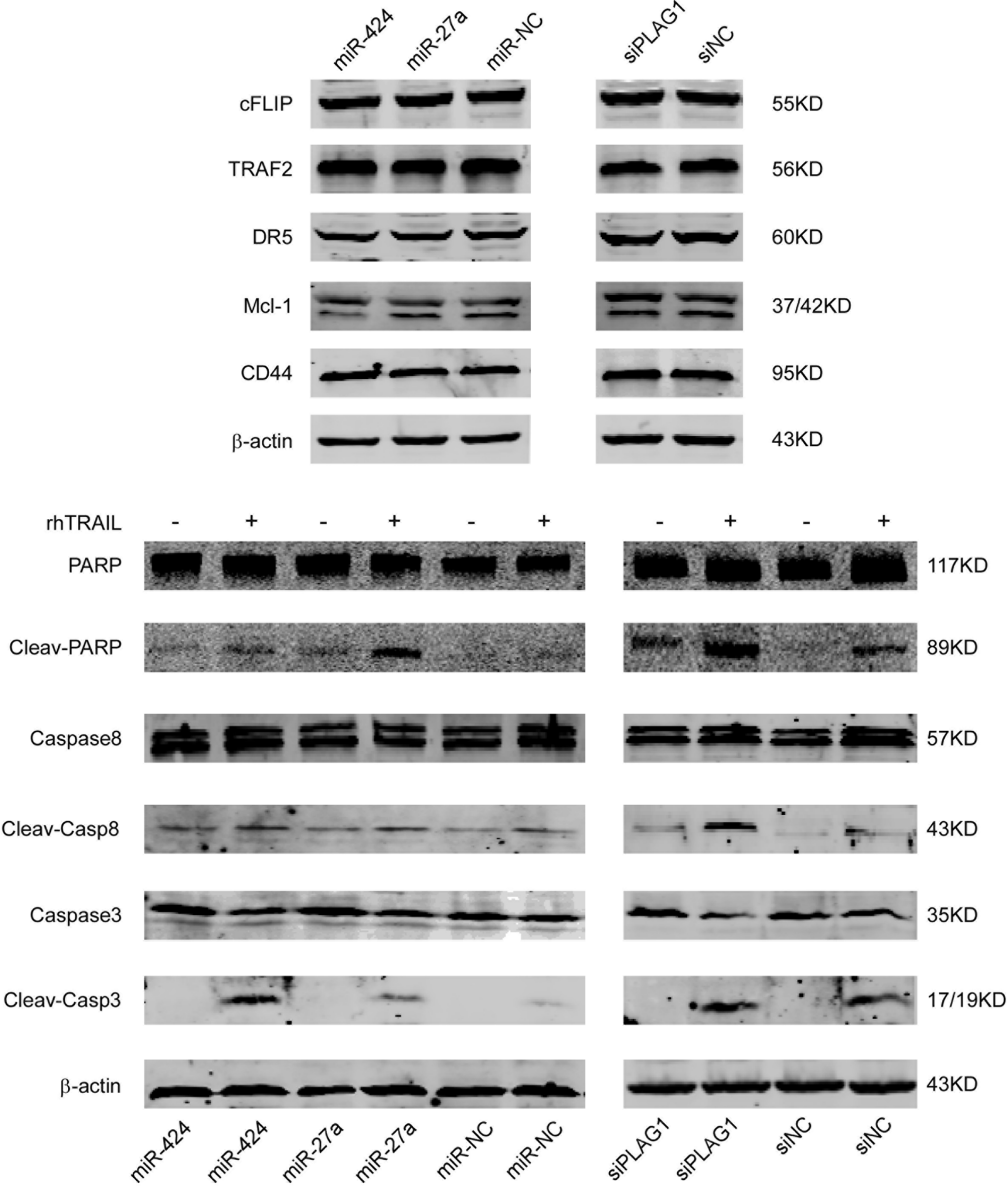
Increased miR-424 and miR-27a or decreased PLAG1 do not regulate DR, Mcl-1 or cFLIP protein levels but augment the cleavage of Caspase8, Caspase3, PARP evidently (**A**) Expression of the indicated proteins was examined in PLAG1 knockdown or miR-424, miR-27a overexpressing cells, and in their respective controls, by Western Blot analysis. The blot for β actin was performed to confirm equal loading of samples. (**B**) miR-424, miR-27a overexpressing or PLAG1 knockdown K562 cells were treated with (+) or without (−) 200 ng/ml TRAIL for 3 h and then analyzed by Western Blot for the indicated proteins. Cleav-PARP, Cleaved PARP; Cleav-Casp8, Cleaved Caspase8; Cleav-Casp3, Cleaved Caspase3.

**Figure 6:**
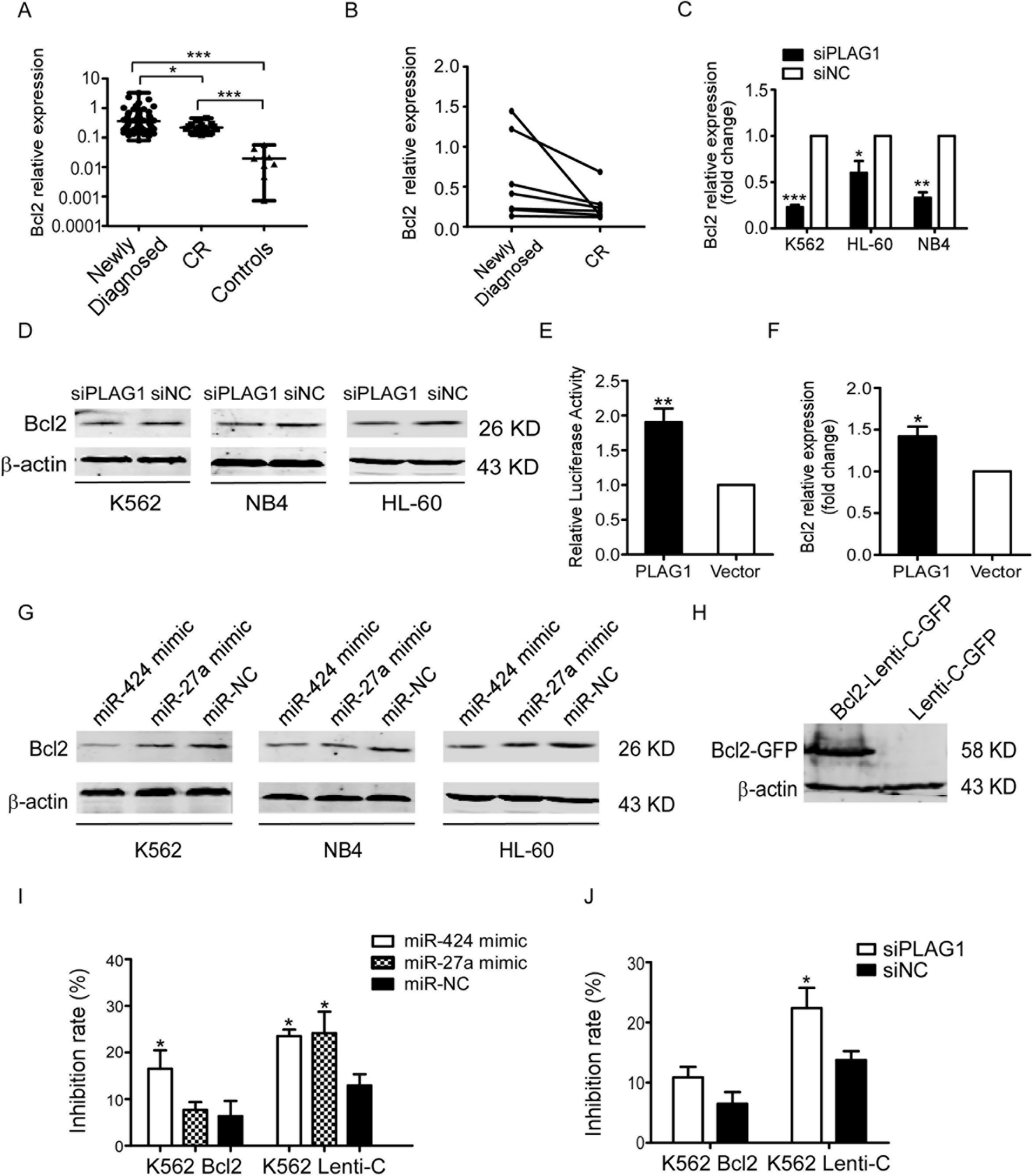
Inhibition of TRAIL-induced apoptosis depends on the transcriptional activation of Bcl2 aroused by PLAG1 upregulation (**A**) qRT-PCR detected expression of Bcl2 in 62 newly diagnosed AML patients, 16 CR patients and 9 normal controls. Solid points indicate individual values and horizontal lines represent medians with range. *p < 0.05, ***p < 0.001. (**B**) qRT-PCR demonstrated that Bcl2 mRNA expression in the BM samples decreased when AML patients achieved complete remission compared with that of the samples obtained from the same patients at the time of diagnosis. (**C-D**) qRT-PCR and Western Blot analysis examined Bcl2 expression after 48 h of transfection with siPLAG1 and negative control. (**E**) Dual luciferase assays presented that PLAG1 overexpression enhanced Bcl2 promoter activity significantly in 293T cells. **p < 0.01. (**F**) Bcl2 mRNA expression in 293T cells increased significantly after 48 h of transfection with p3xFLAG-CMV-10-PLAG1 plasmid. *p < 0.05. (**G**) Bcl2 protein expression was assessed by Western Blot after 48 h of transfection with miR-424 mimic, miR-27a mimic or negative control. (**H**) Western Blot analysis validated Bcl2 overexpression in K562 cells stably infected with Bcl2-Lenti-C-GFP. (**I–J**) K562 cells stably expressing Bcl2-Lenti-C-GFP were transfected with either miR-424 and miR-27a or siPLAG1 and their respective controls. 48 h later cells were treated with or without 200 ng/ml TRAIL for an additional 48 h. Cell viability was quantified with the CCK8 assays. Take the drug free group as control to calculate the inhibition rate of cell growth. *p < 0.05.

To identify the mechanisms implicated in TRAIL resistance, we examined the protein expression of several classical molecules that have been reported to regulate TRAIL-induced apoptosis. The protein levels of DR5, Mcl-1, cFlip, TRAF2 and CD44 that are known to favor or counteract TRAIL-induced cell death remained unchanged after miR-424&27a upregulation or PLAG1 downregulation (Figure 5, above). Anti-apoptotic protein Bcl2 expression was found negatively correlated with miR-424 and miR-27a while positively associated with PLAG1 expression (Figure 6D, 6G). Furthermore, we detected strengthened processing of several executors downstream TRAIL-induced apoptosis in K562 cells upon transfection with either miR-424/27a mimic or siPLAG1. As shown in Figure 5 below, increased miR-424 and miR-27a facilitated processing of Caspase8, Caspase3 and poly(ADP-ribose)ploymerase (PARP) in response to TRAIL treatment. PLAG1 silencing by siRNA also augmented cleavage of Caspase8, Caspase3 and PARP, indicating that miR-424 and miR-27a might enhance TRAIL sensitivity by targeting PLAG1 through activating apoptotic pathways.

### PLAG1 transcriptionally activates Bcl2 in AML

Concurrent with PLAG1, Bcl2 was evidently upregulated in the newly diagnosed AML patients versus CR patients as well as controls (Figure 6A). Similarly, Bcl2 expression of newly diagnosed AML patients dropped down from a relatively higher level when they achieved CR state (Figure 6B). Then we inquired whether Bcl2 expression was manipulated by PLAG1 in AML cells. It was demonstrated that PLAG1 silencing strongly attenuated Bcl2 expression at both mRNA and protein levels (Figure 6C–6D).

To determine how PLAG1 could regulate Bcl2 expression, we constructed 3xFLAG-CMV-10-PLAG1 plasmid and tranfected it into 293T cells. 48 h after transfection, qRT-PCR confirmed that PLAG1 upregulation caused about 41.9% elevation of Bcl2 mRNA expression (Figure 6F). Cotransfection of 3xFLAG-CMV-10-PLAG1 and LB322, a luciferase reporter vector containing full length of Bcl2 promoter, into 293T cells increased Bcl2 promoter activity by up to 90% (Figure 6E). We also found that transfection of AML cells with miR-424 and miR-27a mimic resulted in Bcl2 downregulation (Figure 6G).

We next addressed the role of Bcl2 in AML TRAIL resistance. Forced expression of Bcl2 in K562 cells was verified by Western Blot (Figure 6H). As presented in Figure 6I, upregulation of Bcl2 partially rescued the proliferation inhibition aroused by miR-424 and miR-27a mimic transfection in the presence of TRAIL. Likewise, overexpressed Bcl2 also impaired the TRAIL sensitizing effects resulting from PLAG1 downregulation (Figure 6J). In consequence, these data demonstrate that miR-424 and miR-27a sensitized AML cells to TRAIL by Bcl2 transcriptional repression via targeting PLAG1 directly.

## DISCUSSION

Accumulating evidence show that either innate or acquired TRAIL resistance of cancer cells could be reverted by molecular intervention, among which miRNAs are regarded as effective regulators of TRAIL-induced cell death. So far the precise mechanisms through which miRNAs get involved in AML TRAIL resistance remain unclear. In this study, we presented that miR-424 and miR-27a were downregulated in TRAIL-resistant AML cells. Interestingly, TRAIL sensitivity of the multidrug resistant (MDR) variants K562/A02 and HL-60/ADM was significantly higher than their parental cell lines. One of the most frequently described mechanisms of MDR is the overexpression of P-glycoprotein (P-gp), a ATP-dependent transmembrane protein which is able to pump numerous chemotherapeutic drugs out of cells. Soo-Jung et al. reported that P-gp could enhance TRAIL sensitivity of MDR cancer cells by interacting with DR5 [27]. P-gp expression was increased in K562/A02 and HL-60/ADM cells [28], which may in turn sensitize them to TRAIL-induced apoptosis. Coincidentally, augmented TRAIL sensitivity was found in cisplatin-resistant cancer cell lines due to redistribution of DR4 and various components of the death inducing signaling complex (DISC) to the lipid raft subdomains of plasma membrane [29]. Tumors gained chemotherapy resistance after continuous exposure to a single class of drugs. During this process, the rewiring of oncogenic signaling networks could possibly change and enforce other apoptotic pathways as well.

By proliferation and apoptosis assays, we unveiled that overexpression of miR-424&27a enhanced TRAIL sensitivity of AML cells. Studies have clarified that miR-424&27a were involved in chemoresistance and pathogenesis of hematopoietic malignancies. MiR-424 expression was notably decreased in CML cell lines and patient samples at time of diagnosis while restoration of miR-424 sensitized CML cells to imatinib treatment [30]. Han BW et al. also proposed that overexpression of miR-27a was associated with individual relapse free survival in childhood acute lymphocytic leukemia (ALL) [31]. Downregulated miR-27a was discovered to arouse chemotherapy resistance and relapse in AML [32]. However, in this research we identified for the first time that miR-424 and miR-27a exert effective functions on improving AML TRAIL sensitivity.

To caste insight into the underlying molecular mechanisms by which miR-424 and miR-27a influence AML TRAIL sensitivity, we used bioinformatic methods to predict their possible target genes. As shown in TargetScan, PLAG1 is the common target of miR-424 and miR-27a. By luciferase assays we further confirmed that miR-424 and miR-27a could directly target 3′UTR of PLAG1. PLAG1 was initially discovered by studying the t(3,8) (p21, q12) chromosome translocation, which recurrently occurs in human pleomorphic adenomas of the salivary glands [33]. PLAG1 is generally considered as an oncogene in multiple human tumors, such as lipoblastoma [34], hepatoblastoma [35, 36], chronic lymphocytic leukemia [23], acute myeloid leukemia [37], etc. It has been proved to be the direct target of miR-424 based on the luciferase reporter assays and its overexpression was putatively involved in CLL pathogenesis [23]. Moreover, PLAG1 and PLAGL2 might participate in AML pathogenesis by cooperating independently with fusion protein CBF beta-SMMHC to block hematopoietic differentiation and by promoting proliferation with expanded hematopoietic progenitors [37]. As a transcription factor, PLAG1 exerts its oncogenic potential via cellular signaling triggered by its downstream targets [38]. Nonetheless, researches concerning the specific roles that PLAG1 plays in AML TRAIL resistance have not been reported.

In this study, we presented that PLAG1 expression was increased in TRAIL-resistant AML cells. We also detected higher level of PLAG1 expression in the bone marrow samples obtained at the time of diagnosis than that of the samples from the same patients acquired after complete remission. PLAG1 mRNA expression was negatively correlated with that of miR-424 and miR-27a in AML cell lines as well as AML samples. However, enforced miR-424 and miR-27a attenuated endogenous PLAG1 expression virtually at protein level with marginal change of its mRNA expression. This is probably because most mammalian miRNAs target genes are regulated by translation repression rather than mRNA degradation [23]. In addition, *in vitro* experiments verified that PLAG1 silencing enhanced efficacy of TRAIL treatment for AML cells. Based on the evidence mentioned above, we concluded that miR-424 and miR-27a could reinforce AML sensitivity to TRAIL by targeting PLAG1 post-transcriptionally.

It has been reported that primary AML blasts [40–42] and AML cell lines including K562 [43], HL-60 [42], NB4 [43] are largely resistant to TRAIL-induced apoptosis. Several mechanisms involved in AML TRAIL resistance have been outlined, such as decreased expression of DR4 and DR5, increased expression of DcR1 and 2 which are unable to evoke apoptosis upon TRAIL binding, upregulaion of cFLIP that blocks the recruitment of Caspase8 to DISC, accumulation of Mcl-1 and other anti-apoptotic proteins that belong to Bcl2 family. As assessed by Western Blot, neither elevated miRNAs expression nor PLAG1 knockdown altered DR4 (data not shown), DR5, cFLIP and Mcl-1 expression, which implicated that miR-424 and miR-27a did not regulate TRAIL sensitivity by modulating the above molecules.

Bcl2 has been previously shown to confer TRAIL resistance to leukemia cells [44]. We observed that upon transfection with miR-424/27a mimic or PLAG1 siRNA, TRAIL-induced apoptosis was potentiated along with augmented cleavage of Caspase8, Caspase3, PARP and reduced expression of Bcl2. Intriguingly, Bcl2 expression altered concurrently with PLAG1 in AML cell lines and AML patients. Moreover, our data suggested that PLAG1 upregulation enhanced Bcl2 promoter activity and therefore reinforced its transcription. Similarly, Voz et al. identified Bcl2 as a possible downstream target of PLAG1 since it was consistently upregulated in PLAG1-expressing cells and the tissues of pleomorphic adenomas of the salivary glands [45]. Furthermore, we revealed that miR-424 and miR-27a negatively modulated Bcl2 expression in AML cells. Increased Bcl2 expression abrogated the TRAIL sensitizing effects of miR-424, miR-27a and PLAG1 silencing in K562 cells. Thus, based on these results, we concluded that miR-424&27a downregulate Bcl2 by targeting PLAG1.

In summary, these data highlight a mechanism, involving PLAG1, through which miR-424 and miR-27a enhance AML sensitivity to TRAIL. It is noteworthy that PLAG1 exerts its anti-apoptotic functions on AML cells by transcriptional activation of Bcl2 which interferes with both intrinsic and extrinsic apoptotic pathways via cleavage blockade of Caspase8, Caspase3 and PARP. Thus approaches, targeting miR-424&27a and/or PLAG1 could be potentially of value in AML TRAIL therapy.

## MATERIALS AND METHODS

### TRAIL

Human recombinant TRAIL/Apo2L was bought from Peprotech, USA and dissolved in 0.1% BSA (Sigma-Aldrich, St Louis, MO). After full dissolution, it was splited into small packages and stored at −80°C.

### Patient samples

After informed consent, bone marrow (BM) samples were obtained from 78 AML patients and 10 healthy donors at Qilu Hospital, Shandong University. The study protocols were approved by the Medical Ethical Committee of Qilu Hospital, Shandong University. Mononuclear cells were isolated from the bone marrow samples using density-gradient centrifugation with Ficoll-Paque Plus (Ficoll, Pharmacia LKB Biotechnology, Piscataway, NY) and stored at −80°C. We classified AML patients into newly diagnosed group and complete remission group. Several matched-pair BM samples were acquired from the same patients both at the time of diagnosis and at the complete remission state. Detailed clinical information for AML patients is available in Table 1.

**Table 1:** Characteristics of 78 AML patients

Variables	No. of patients	
	Newly-diagnosed	CR
No. of patients	62	16
Sex (male/female)	37/25	8/8
Age: median (range)	34 (12–76)	29 (18–44)
FAB Classification		
M0	1	0
M1	1	0
M2	12	5
M3	16	9
M4	14	1
M5	18	1
M6	0	0
Hemoglobin (g/l): median (range)	78 (46.8–123)	104 (78–130)
WBC (×109/l): median (range)	15.73 (0.8–583.9)	3.73 (2.27–34.85)
Neutrophils (%): median (range)	22.4 (0.1–86)	58.7 (39.2–85.3)
Lymphocytes (%): median (range)	27.2 (0.0–96.9)	26.2 (5.9–48)
PLT (×109/l): median (range)	33 (4–315)	326 (119–916)

Abbreviations: FAB, French-American-British classification; WBC, white blood cells; PLT, platelets.

### Cells and cell culture

Human AML cell lines HL-60, HL-60/ADM, K562, K562/A02 and NB4 were derived from Institute of Hematology & Blood Diseases Hospital Chinese Academy of Medical Sciences & Peking Union Medical College (Tianjin, China). Except for HL-60, cells were cultured by RMPI-1640 (Hyclone, Logan, UT, USA) plus 10% fetal bovine serum (TBD, Haoyang, China) in a 37°C humidified incubator with 5% CO_2_. HL-60 was particularly cultivated in IMDM (Hyclone, Logan, UT, USA) supplemented with 20% fetal bovine serum (Gibco, Life, USA).

### Proliferation assay

Concisely, cells with or without transfection of miRNAs or siRNAs were cultured in 96-well plates and treated with serial dilutions of TRAIL (Peprotech, USA) at concentrations of 0, 20, 50, 100, 200 ng/ml for 48 h. Cell Counting 8-assay (Beyotime, China, JS) was added to each well and cells were incubated for 4 h at 37°C. The absorbance was measured at 570 nm and 670 nm simultaneously and we calculated inhibition rate of cell growth and IC_50_ values (the concentration of drug at which 50% of cell proliferation inhibited). Each test was performed in triplicates.

### *In vitro* transfection with synthetic miRNAs or siRNAs

The synthetic mimics of miR-424 and miR-27a along with scrambled oligonucleotides were purchased from GenePharma (Shanghai, GenePharma Co.Ltd). SiRNAs of PLAG1 and its negative control were synthesized by Ribobio (Guangzhou Ribo Bio Co. Ltd). Lipofectamine 2000 reagent (Invitrogen, Carlsbad, CA) was used to transfect cells with miRNAs or siRNAs at a final concentration of 50 nM according to the manufacturer's instructions.

### Apoptosis assay

The apoptosis assay was conducted using Annexin V/propidium iodide (PI) Apoptosis Detection Kit (Invitrogen, Carlsbad, CA) under the guidence of the manufacturer's instructions. Apoptosis rate of cells was measured by Becton Dickinson FACS Calibur flow cytometer, detecting the fluorescence of at least 10000 cells each sample.

### Quantitative real-time PCR

Total cellular RNA was extracted from cell lines using Trizol (Invitrogen, Carlsbad, CA, USA) according to the manufacturer's protocols. TaqMan MicroRNA Assay (ABI, Foster City, CA, USA) was used to detect the expression levels of mature miR-424 and miR-27a. As for PLAG1, Bcl2 expression, quantitative real-time PCR was performed by a Applied Biosystem 7900HT System (ABI, Foster City, CA, USA) using SYBR Green PCR Master Mix (Toyobo, Osaka, Japan). Each sample was run in triplicates and amplified in a 20 μl reaction volume under the manufacturer's instructions. The house-keeping gene GAPDH, which has relatively constant expression level in AML cell lines, was used as an internal control. The primers for real time quantification are listed in Table 2.

**Table 2:** Primer sets and genes included in qPCR

Name	Forward primer	Reverse primer
PLAG1	5′-CCGGTTCTACACCCGAAAGG-3′	5′-GCACAATACTGACAGAGGAAGTC-3′
Bcl2	5′-GGTGGGGTCATGTGTGTGG-3′	5′-CGGTTCAGGTACTCAGTCATCC-3′
GAPDH	5′-GGAGCGAGATCCCTCCAAAAT-3′	5′-GGCTGTTGTCATACTTCTCATGG-3′

### Western blot

Harvested cells were fully resolved by incubating in ice-cold radioimmunoprecipitation assay buffer (RIPA) with protease and phosphatase inhibitors. Afterwards proteins were fractionated by SDS-PAGE and transferred onto nitrocellulose membranes (Millipore, Billerica, MA). Immunoreactive bands were detected and quantitated with the LI-COR Infrared imaging system (Lincoln, NE). β-actin was served as a loading control.

### Plasmids and lentiviral particles construction

For stable expression of PLAG1, full length of its open reading frame (ORF) was PCR-amplified from human genomic cDNA and inserted between the KpnI and XbaI sites of p3xFLAG-CMV-10 vector (kindly provided by Dr Xiulian Sun, Qilu Hospital of Shandong University). The upstream and downstream amplification primers are 5′-GGG GTA CCA ATG GCC ACT GTC-3′ and 5′-GCT CTA GAC TGA AAA GCT TGA TG-3′ respectively. Similarly the ORF of Bcl2 cloned from Bcl2-pCEP4 (Addgene, Plasmid# 16461) was inserted into pLenti-C-GFP vector. PCR primers are as follows:5′-ATA TGC GAT CGC CAT GGC GCA CGC T-3′ and 5′-CGA CGC GTC TTG TGG CCC AG-3′. To package Bcl2-Lenti-C-GFP or control lentiviral particles, Hek293T cells were co-transfected with a mixture of 10 μg pLenti-C-GFP-Bcl2 or pLenti-C-GFP, and 6.67 μg psPAX2, 3.3 μg pMD2.G utilizing Lipofectamine 2000 transfection reagent (Invitrogen, Carlsbad, CA).

### Luciferase reporter assay

Full length of the 3′UTR of PLAG1 with or without mutations was cloned from human genomic cDNA and inserted into pMIR-REPORT vector (Ambion Inc., Austin, TX, USA) upstream the firefly luciferase coding sequence, which formed a construct carrying the wildtype 3′UTR (pPLAG1_WT) and constructs carrying 3′UTR sequences with mutations at the putative recognition sites of miR-424 and miR-27a (pPLAG1_424M and pPLAG1_27aM respectively). Wildtype and mutant inserts were confirmed by DNA sequencing. 293T cells were cotransfected with 0.5 ug reporter gene constructs and miR-424 mimic, miR-27a mimic or scrambled control using Lipofectamine 2000 (Invitrogen, Carlsbad, CA). Then firefly and renilla luciferase activities were measured by the dual-luciferase reporter assay system (Promega) under the manufacturer's instructions.

Plasmid LB322 containing Bcl2 promoter from ATG to -3934 (Addgene, Plasmid#15381) and p3xFLAG-CMV-10-PLAG1 were co-transfected into 293T cells and we conducted dual-luciferase reporter assay as mentioned above.

### Statistical analysis

Each experiment was performed at least three times and all the data were presented as mean ± S.E. Comparison between control and experimental groups was conducted using Student's *t*-test. To measure the discrepancy between clinical AML samples, Mann-Whitney test was used. All the tests were two tailed and computed by SPSS (version 17.0) software. *P* < 0.05 was considered statistically significant.
